# A Case of Philadelphia Chromosome Positive Myeloproliferative Neoplasm in a Pregnant Woman with Unusual Primary Myelofibrosis Features

**DOI:** 10.1155/2013/702831

**Published:** 2013-05-25

**Authors:** Jason Koshy, Jack Alperin, Bagi Jana, Avi Markowitz, You-Wen Qian

**Affiliations:** ^1^Division of Hematopathology, Department of Pathology and Laboratory Medicine, University of Texas Medical Branch, 301 University Boulevard, Galveston, TX 77555, USA; ^2^Division of Hematology and Oncology, Department of Internal Medicine, University of Texas Medical Branch, 301 University Boulevard, Galveston, TX 77555, USA

## Abstract

Myeloproliferative neoplasms (MPNs) are traditionally separated into *BCR-ABL*-positive chronic myeloid leukemia (CML), and BCR-ABL-negative MPNs including primary myelofibrosis (PMF), essential thrombocythemia (ET), and so forth. One of the diagnostic requirements for PMF and ET is the absence of the Philadelphia chromosome, while its presence is almost universally indicative of CML. However, a diagnostic dilemma arises when Philadelphia chromosome-positive MPNs lack the majority of the typical features seen in CML. Some of these classic CML features include basophilIa, marked leukocytosis, neutrophils left-shift with myelocytes bulge, and “dwarf” megakaryocytes. Presented here is a case of a 32-year-old pregnant patient who did not have typical morphologic findings for CML, and yet the Philadelphia chromosome was positive. The patient demonstrated some pathologic features that are commonly presented in PMF that included bone marrow reticulin fibrosis, leukoerythroblastosis, splenomegaly, and increased serum lactate dehydrogenase.

## 1. Introduction

Primary myelofibrosis (PMF) is a distinct, well-defined myeloproliferative neoplasm (MPN) characterized by megakaryocytic hyperplasia and subsequent fibrosis in the bone marrow, as well as leukoerythroblastosis and organomegaly. The presence of the *t*(9; 22), also known as the Philadelphia chromosome translocation, is used to demarcate chronic myelogenous leukemia (CML) from other MPNs including PMF. We describe an unusual case of a 32-year-old pregnant patient who presented with thrombocytosis and was found to have Philadelphia chromosome. The overall clinical presentation in this patient including bone marrow features were more commonly seen in a PMF, although other MPNs such as essential thrombocythemia (ET) were also considered.

## 2. Case Presentation

**Figure 1:**
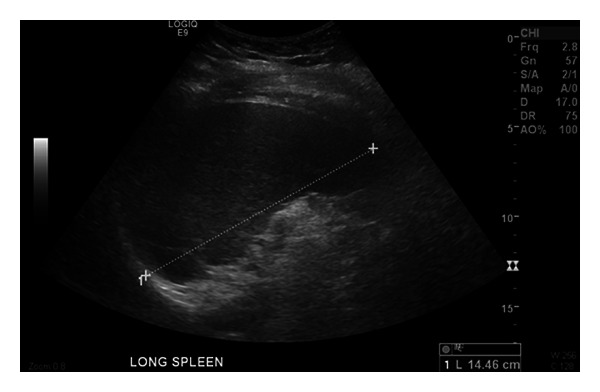
Abdominal ultrasonogram showing the patient's splenomegaly.

**Figure 2:**
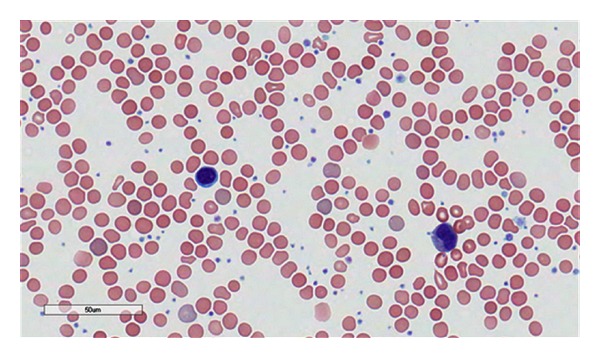
Peripheral smear showing leukoerythroblastosis and thrombocytosis.

**Figure 3:**
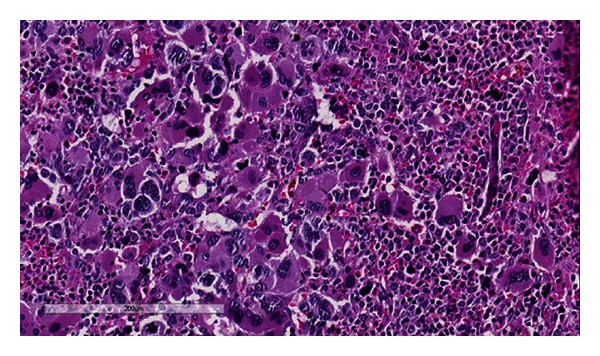
H & E section showing a markedly hypercellular bone marrow with megakaryocytic hyperplasia.

**Figure 4:**
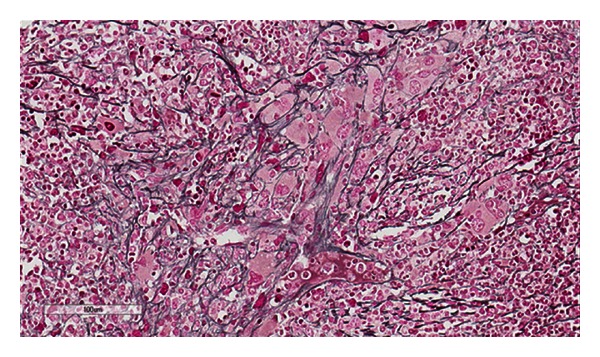
Reticulin stain of bone marrow showing 2+ fibrosis.

The patient was a 32-year-old gravid 2 para 1 patient at 8 weeks gestation who presented to her obstetrician for diabetes management. She had a history of thrombocytosis in 2010 and was prescribed Plavix by her physician at an outside hospital. She denied any history of thrombosis but did complain of bleeding when she brushed her teeth. The physical examination was significant for hepatosplenomegaly which was later confirmed with abdominal imaging (Figure 1). Initial complete blood count (CBC) revealed a platelet count of 1,291 × 10^9^/L (reference range 166–358 × 10^9^/L), hemoglobin of 10.7 g/dL (reference range 11.6–15 g/dL), hematocrit 33.4% (reference range 35.7–45.2%), and a white blood cell (WBC) count of 15.7 × 10^9^/L (reference range 4.3–11.1 × 10^9^/L) with no basophilia. The lactate dehydrogenase (LDH) level measured 679 U/L (reference range 300–600 U/L). A peripheral blood smear review (Figure 2) confirmed the marked thrombocytosis which included atypical platelet morphology such as the presence of megathrombocytes, giant platelets, and hypogranulated platelets. The blood smear also revealed numerous nucleated red blood cells and a few immature neutrophils consistent with leukoerythroblastosis. No dysplastic changes were appreciated in any cell line. A bone marrow biopsy was performed that showed remarkable megakaryocytic hyperplasia with many tight megakaryocytes clusters. Most megakaryocytes had abundant cytoplasm and hyperlobation. There were only a few megakaryocytes with hyperchromatic nuclei. “Dwarf” megakaryocytes were infrequent (Figure 3). There was also mild myeloid hyperplasia with 2+ reticulin fibrosis (Figure 4). 

A fresh sample of the bone marrow aspirate was collected for chromosome and FISH analysis. Cytogenetic analysis of CTG banded metaphases revealed the presence a karyotype of 46, XX, *t*(9,22)(q34, q11.2). The FISH study on the sample received was positive for the *BCR/ABL* gene involvement in 37% of interphase cells analyzed using unique sequence dual fusion DNA probes for the *BCR* (22q11.2) and *ABL1* (9q34) loci and a control gene argininosuccinate synthetase (*ASS*) located adjacent to ABL1 (Vysis Inc., Des Plains, IL, USA).

Patient's DNA was isolated from blood and subjected to allele-specific PCR amplification. The reaction used an oligonucleotide primer set specific for the exon 14 of *JAK2* on chromosome 9 and an allele-specific primer that specifically initiates amplification from the allele containing the point mutation in codon 617. This allele-specific PCR amplification failed to detect the JAK2 mutation. RNA was isolated from bone marrow and reverse transcribed. The resulting cDNA was subjected to separate PCR amplifications with primers designed to amplify fusions that will give rise to either the p190 or p210 forms of *BCR*-*ABL1*. An additional RT-PCR amplification was directed at the *ABL1* gene as a control for sample quality. The PCR products were resolved by electrophoresis and evaluated for the presence of amplicons that indicate a positive result. Qualitative RT-PCR detected the major *BCR/ABL* translocation while BCR-ABL/ABL quantitative ratio was 0.36657 with an International Scale (percent) of 30.6789.

The above cytogenetics, FISH analysis, and BCR-ABL studies were performed at ARUP laboratories, Salt Lake City, UT, USA. 

The patient was discharged on a daily hydroxyurea and aspirin regimen with a goal of lowering her platelet count. Serial CBCs after her discharge failed to show a significant decrease in the platelet count. The leukocytosis remained only mild with negligible myelocytosis, and basophilia was again absent. The patient was given imatinib after cesarean section.

## 3. Discussion

The clinical presentation, bone marrow histologic findings, and molecular study results seen in our patient raised the differential diagnoses of PMF, ET, and CML. Despite the fact that a positive Philadelphia chromosome translocation has been reported in 85% of all cases of CML [1], there were multiple findings in this patient that argued against a diagnosis of CML. Firstly, absolute basophilia, which is almost always seen in patients with CML, was not detected in our patient. Secondly, there was only a minimal leukocytosis and just a nominal increase in myelocytes. Classic CML characteristically has a marked increase in both the total WBC count (median 170 × 10^9^/L) and immature neutrophil precursors [2]. Finally, “dwarf” megakaryocytes, which are smaller than normal megakaryocytes and have hypolobated nuclei, are usually associated with CML but were a rare finding in the bone marrow biopsy in our patient. 

ET was in the differential diagnosis due to the marked thrombocytosis. However, the presence of leukoerythroblastosis, a hypercellular bone marrow with reticulin fibrosis, the myeloid hyperplasia, and the megakaryocytic morphology as well as the absence of the *JAK2 *mutation did not favor a diagnosis of ET. Of note, Philadelphia chromosome translocation has been rarely reported in ET. Mishra et al. described an 8-year-old girl with asymptomatic thrombocytosis and Philadelphia chromosome positivity [3]. The thrombocytosis in that patient was refractory to imatinib alone and required the addition of hydroxyurea. CML patients may also present clinically similar to ET [4]. 

In our patient, there were multiple clinical and histologic findings that are commonly seen in PMF. The overall cellularity of the marrow was markedly increased with a cell to fat ratio of 9 : 1. The bone marrow showed remarkable megakaryocytic hyperplasia with many tight clusters. These findings are typically found in the prefibrotic or early stage of PMF. Interestingly, the reticulin stain showed moderate (2+) fibrosis. Although this megakaryocytic hyperplasia and marrow reticulin fibrosis are not specific for PMF, our case fills all four of the WHO-defined minor criteria for PMF which include leukoerythroblastosis, increased serum LDH, anemia, and splenomegaly. However, according to the current WHO, the presence of BCR-ABL translocation precludes the diagnosis of PMF. 

PMF is the least prevalent of all MPNs in women of child-bearing age and generally has a poorer prognosis than other MPNs in this age group [5] due to complications of thrombosis in the mother as well as in the placental circulation [6]. The presence of *t*(9; 22) complicates the final diagnosis in this case as this translocation is predominantly associated with CML. There have been only rare reports of Philadelphia chromosome-positive PMF, the latest by Chen et al. in 2007 [7]. Their patient presented in the overtly fibrotic phase of PMF and showed no features of CML in the bone marrow or peripheral blood. Similar to our case, their patient had leukoerythroblastosis, anemia, splenomegaly, and a negative *JAK2* mutation. Despite the presence of the typical *BCR*/*ABL* translocation, no obvious response to imatinib was seen. It should also be noted that there have been reports of *BCR*/*ABL* fusion genes in leukocytes of individuals with no evidence of leukemia [8]. 

One potential explanation for the findings in our patient is the possibility of one MPN masking the presence of another. There have been reports of coexisting MPNs involving CML [9, 10]. However, PMF had never been reported as one of the coexisting MPNs with CML until 2009 when Laibe et al. published a case of a patient who initially presented with PMF but developed CML 7 years later [11]. Retrospective FISH analysis detected the two clones of CML and PMF as evidenced by the distinct and separate presence of both *t*(9; 22) and 13q deletion, respectively. Treatment with imatinib caused the disappearance of the CML clone and an increase in the PMF clone. This suggests that the CML clone may have been suppressing the PMF clone. In our patient, the only cytogenetic abnormality was *t*(9; 22). Though it is possible that PMF may be hiding CML in our patient, it is difficult to fully assess due to the lack of two distinct clonal populations. The other explanation might be the evolution of a pre-existing MPN into CML or the existence of a common stem cell as the target of an early initiating event (such as a mutation) leading to chromosomal instability and favoring the development of 2 MPNs (clonal evolution).

To date, no specific genetic abnormality has been directly linked with PMF, though 33–45% of cases have some type of clonal karyotype anomaly [12]. The presence of the *JAK2 *mutation or other clonal marker is helpful for diagnosis, but only approximately 50% of PMF-affected patients are positive for the *JAK2* mutation [13]. Indeed, our patient did not carry the *JAK2 *mutation by PCR analysis of a peripheral blood sample. However, the significance of the *JAK2* mutation in PMF is questionable as it relates to prognosis. One JAK inhibitor, ruxolitinib, was shown to be equally effective in patients with or without mutated *JAK2 *[14]. While there has been marginal to absent survival benefit seen with the current JAK inhibitors, there has been a significant improvement of constitutional symptoms and a decrease of spleen size.

While the differential diagnosis remains, the clinical responses to the imatinib are being closely monitored. Pregnant patients with MPNs present a difficult problem for the treating obstetrician and hematologist. Normal pregnant women are six times more likely to have thrombotic complications compared to a nonpregnant woman, and this risk is multiplied greatly if an MPN is also present [15]. This makes timely and accurate diagnosis of an MPN critical in maternal-fetal care. The presence of the Philadelphia chromosome translocation while the marrow feature being consistent with PMF makes this case unique, which further complicates clinical management of such patients.
